# Denosumab and the Risk of Diabetes in Patients Treated for Osteoporosis

**DOI:** 10.1001/jamanetworkopen.2023.54734

**Published:** 2024-02-09

**Authors:** Huei-Kai Huang, Albert Tzu-Ming Chuang, Tzu-Chi Liao, Shih-Chieh Shao, Peter Pin-Sung Liu, Yu-Kang Tu, Edward Chia-Cheng Lai

**Affiliations:** 1 Department of Family Medicine and Department of Medical Research, Hualien Tzu Chi Hospital, Buddhist Tzu Chi Medical Foundation, Hualien, Taiwan; 2 School of Medicine, Tzu Chi University, Hualien, Taiwan; 3 Institute of Epidemiology and Preventive Medicine, College of Public Health, National Taiwan University, Taipei, Taiwan; 4 School of Pharmacy, Institute of Clinical Pharmacy and Pharmaceutical Sciences, College of Medicine, National Cheng Kung University, Tainan, Taiwan; 5 Department of Pharmacy, Keelung Chang Gung Memorial Hospital, Keelung, Taiwan; 6 Center for Healthy Longevity, Hualien Tzu Chi Hospital, Buddhist Tzu Chi Medical Foundation, Hualien, Taiwan; 7 Institute of Medical Sciences, Tzu Chi University, Hualien, Taiwan; 8 Department of Dentistry, National Taiwan University Hospital and School of Dentistry, National Taiwan University, Taipei, Taiwan; 9 Institute of Health Data Analytics and Statistics, College of Public Health, National Taiwan University, Taipei, Taiwan

## Abstract

**Importance:**

Denosumab, a humanized monoclonal antibody against receptor activator of nuclear factor κB ligand (RANKL), is a widely used antiresorptive medication for osteoporosis treatment. Recent preclinical studies indicate that inhibition of RANKL signaling improves insulin sensitivity, glucose tolerance, and β-cell proliferation, suggesting that denosumab may improve glucose homeostasis; however, whether denosumab reduces the risk of incident diabetes remains unclear.

**Objective:**

To evaluate whether denosumab use is associated with a lower risk of developing diabetes in patients with osteoporosis.

**Design, Setting, and Participants:**

This nationwide, propensity score–matched cohort study used administrative data from Taiwan’s National Health Insurance Research Database. Adult patients who received denosumab for osteoporosis therapy in Taiwan between 2012 and 2019 were included. To eliminate the inherent bias from confounding by indication, the patients were categorized into a treatment group (34 255 patients who initiated denosumab treatment and adhered to it) and a comparison group (34 255 patients who initiated denosumab treatment but discontinued it after the initial dose) according to the administration status of the second dose of denosumab. Propensity score matching was performed to balance patient characteristics and to control for confounders.

**Exposure:**

Treatment with denosumab.

**Main Outcomes and Measures:**

The primary outcome was incident diabetes requiring treatment with antidiabetic drugs. A Cox proportional hazards model was used to estimate the hazard ratio (HR) for incident diabetes. Data were analyzed from January 1 to November 30, 2023.

**Results:**

After propensity score matching, 68 510 patients were included (mean [SD] age, 77.7 [9.8] years; 57 762 [84.3%] female). During a mean (SD) follow-up of 1.9 (1.6) years, 2016 patients developed diabetes in the treatment group and 3220 developed diabetes in the comparison group (incidence rate, 35.9 vs 43.6 per 1000 person-years). Compared with the comparison group, denosumab treatment was associated with a lower risk of incident diabetes (HR, 0.84; 95% CI, 0.78-0.90). Several sensitivity analyses also demonstrated similar results of lower diabetes risk associated with denosumab treatment.

**Conclusions and relevance:**

The results from this cohort study indicating that denosumab treatment was associated with lower risk of incident diabetes may help physicians choose an appropriate antiosteoporosis medication for patients with osteoporosis while also considering the risk of diabetes.

## Key Points

**Question:**

Is denosumab associated with a lower risk of developing diabetes?

**Findings:**

In this cohort study of 68 510 adults, continued treatment of denosumab for osteoporosis was associated with significantly lower risk of developing diabetes than discontinued denosumab treatment.

**Meaning:**

The findings of this study may help physicians select an appropriate medication for the treatment of patients with osteoporosis while considering that denosumab was also associated with lowering the incidence of diabetes.

## Teaser

This nationwide cohort study assesses whether denosumab therapy is associated with decreased risk of developing diabetes among adults in Taiwan treated for osteoporosis.

## Introduction

Osteoporosis, characterized by reduced bone density and strength, increases the risk of fractures, disability, and mortality, especially among older adults.^1,2^ With the global aging population, the impact of osteoporosis on health systems is growing.^1,3^ Concurrently, diabetes presents significant health risks and contributes to morbidity and mortality in older adults while also exacerbating bone fragility in patients with osteoporosis, making effective prevention of diabetes in these patients crucial.^4,5,6,7^

Recent studies have suggested an association between bone health and glucose metabolism,^8,9,10^ with denosumab, an antiresorptive medication in the treatment of osteoporosis, showing potential for improving insulin sensitivity and glucose tolerance.^11,12^ However, while some clinical evidence suggests denosumab may be positively associated with glycemic parameters, such as fasting plasma glucose and homeostatic model assessment for insulin resistance, its effect on reducing diabetes risk remains unclear.^13^ A previous post hoc analysis of the FREEDOM trial indicated no statistically significant effect of denosumab in lowering diabetes risk,^14^ but limitations due to potential issues of being underpowered and having a small number of diabetes cases make the findings of the trial inconclusive.^14,15^ In contrast, a recent retrospective cohort study in the UK suggested lower diabetes risk associated with denosumab use compared with bisphosphonate use, despite potential confounding bias by indication.^15^ These inconsistent results and potential limitations underscore the need for more research to clarify the effects of denosumab on diabetes risk.

To address these concerns, we conducted a large-scale cohort study comparing diabetes risk between patients who remained adherent to denosumab treatment through the second dose and patients who discontinued after the first dose. Specifically, this study sought to determine whether the continued use of denosumab, in contrast to its discontinuation, is associated with modifying the risk of developing diabetes. Our approach may help mitigate potential confounding by indication, which is common in observational studies, thus providing more reliable evidence assessing the association between denosumab use and diabetes risk.

## Methods

The protocol for this cohort study was approved by the National Cheng Kung University Institutional Review Board, which also waived the requirement for obtaining informed consent due to the use of retrospective and anonymized data. The study was conducted in accordance with the Strengthening the Reporting of Observational Studies in Epidemiology (STROBE) reporting guideline and The Reporting of Studies Conducted Using Observational Routinely-Collected Health Data (RECORD) Statement.^16,17^

### Data Sources

We conducted a nationwide retrospective cohort study using Taiwan’s National Health Insurance Research Database (NHIRD). The National Health Insurance Program is a mandatory single-payer program administered by Taiwan’s government that covers more than 99% of Taiwan’s population (approximately 23.6 million individuals). With records encompassing demographic data and medical claims for inpatient, outpatient, and emergency care services, the NHIRD reflects the Taiwanese population’s health status and health care use. Diagnoses and procedures before 2016 were described using codes from the *International Classification of Diseases, Ninth Revision, Clinical Modification* (*ICD-9-CM*) system, while those after 2016 were described using the codes from the tenth revision (*ICD-10-CM*). The data sets from the NHIRD we used are detailed in the eAppendix in Supplement 1. Mortality information was obtained by cross-referencing the NHIRD with the Taiwan National Register of Death. Further details on the NHIRD have been published elsewhere.^18,19^

### Study Population

The study population included all adult patients who were new users of denosumab, 60 mg (defined as receipt of ≥1 dose of denosumab without any prior use), for osteoporosis treatment between 2012 and 2019 and had no history of malignant neoplasm, Paget disease, or diabetes that required antidiabetic medication. In Taiwan, the criteria for reimbursement of initiating denosumab treatment for osteoporosis include (1) a T score of −2.5 SDs or lower and a hip or vertebral fracture or (2) a T score between −1.0 SD and −2.5 SD and 2 or more hip or vertebral fractures. Initiation of denosumab does not require the use of other prior osteoporosis medications.^20^

### Exposure, Outcome, and Follow-Up

Subcutaneous administration of denosumab is recommended at 6-month intervals for osteoporosis treatment.^20^ Two distinct patient cohorts were established according to the administration status of the second denosumab dose (see eFigure 1 in Supplement 1 for the study design schema). The treatment group included all patients who received their second dose per the anticipated administration schedule 180 days after the initial dose; an additional 45-day grace period was granted for possible administrative challenges, such as scheduling appointments. The index date (start of the follow-up) was 225 days after the initial dose. The comparison group included patients who did not receive a second dose aligned with the anticipated administration schedule (no dose within 225 days from the initial dose). Similar to the treatment group, the index date for the comparison group was 225 days following the initial dose. Constructing such a comparison group was done to eliminate inherent biases caused by the initial treatment decision and confounding by indication, considering that a single dose of denosumab was not expected to offer a sustained clinical advantage.^21^ A landmark approach was used, uniformly setting the index date as 225 days after the initial dose, ensuring that both groups commenced follow-up at the same baseline time point. This effectively circumvented the issue of immortal time bias often encountered in observational studies.^22,23^

The primary outcome was new-onset diabetes requiring treatment with any antidiabetic drug (Anatomical Therapeutic Chemical code A10). For the treatment group, individuals were followed up from the index date until the occurrence of the study outcome, denosumab discontinuation (ie, 225 days after the last administration), death, or the last date in the database (December 31, 2020), whichever occurred first. For the comparison group, follow-up began from the index date until the occurrence of the study outcome, starting or restarting of any osteoporosis therapy (eg, denosumab, bisphosphonates, raloxifene, teriparatide, or calcitonin preparations), death, or until December 31, 2020.

### Covariates

We retrieved information for several covariates to describe patient characteristics and to control for potential confounding factors, including age, sex, monthly income, urban status of residence, health care use, comorbidities, and medication use, as listed in Table 1. We assessed the covariates within 1 year before the index date and identified them through the outpatient, inpatient, and emergency service records in the NHIRD. The diagnosis and drug codes used to identify the covariates are given in eTable 1 in Supplement 1.

**Table 1.** Baseline Characteristics of Treatment and Comparison Groups After Propensity Score Matching^a^

Characteristic	Patients, No. (%)		Standardized difference
	Treatment (n = 34 255)	Comparison (n = 34 255)	
Age, mean (SD), y	77.7 (9.6)	77.7 (9.9)	0.01
Sex			
Male	5390 (15.7)	5358 (15.6)	0
Female	28 865 (84.3)	28 897 (84.4)	
Year of index date			
2012-2014	4178 (12.2)	4174 (12.2)	0.02
2015-2017	12 175 (35.5)	12 135 (35.4)	
2018-2020	17 902 (52.3)	17 946 (52.4)	
Income, NT$			
≥25 000	8919 (26.0)	8907 (26.0)	0
15 000-24 999	17 002 (49.6)	17 036 (49.7)	
≤14 999	8334 (24.3)	8312 (24.3)	
Urban level of residence			
1 (Most urban)	5965 (17.4)	5966 (17.4)	0
2	5374 (15.7)	5502 (16.1)	
3	2603 (7.6)	2570 (7.5)	
4	1154 (3.4)	1154 (3.4)	
5	18 169 (53.0)	18 105 (52.9)	
6	818 (2.4)	789 (2.3)	
7 (Least urban)	172 (0.5)	169 (0.5)	
Location of residence in Taiwan			
Northern	11 940 (34.9)	12 052 (35.2)	0
Central	6081 (17.8)	6033 (17.6)	
Southern	13 277 (38.8)	13 219 (38.6)	
Eastern	2739 (8.0)	2739 (8.0)	
Offshore islands	218 (0.6)	212 (0.6)	
Healthcare use, mean (SD)			
Emergency department visits	1.0 (1.9)	1.0 (1.7)	0
Outpatient visits	40.8 (23.1)	40.2 (25.2)	0.02
Hospitalizations	0.7 (1.1)	0.7 (1.1)	−0.01
Fracture history			
Hip fracture	5118 (14.9)	5061 (14.8)	0
Vertebral fracture	17 323 (50.6)	17 416 (50.8)	−0.01
Wrist or humerus fracture	1688 (4.9)	1690 (4.9)	0
Comorbidity			
Asthma	4958 (14.5)	4841 (14.1)	0.01
Cataract	6434 (18.8)	6497 (19.0)	0
Congestive heart failure	2933 (8.6)	2867 (8.4)	0.01
COPD	6168 (18.0)	6081 (17.8)	0.01
Dementia	4209 (12.3)	4210 (12.3)	0
Depression	1604 (4.7)	1558 (4.6)	0.01
Dyslipidemia	8108 (23.7)	8222 (24.0)	−0.01
Gastrointestinal tract bleeding	1516 (4.4)	1508 (4.4)	0
Glaucoma	1832 (5.4)	1834 (5.4)	0
Gout	4064 (11.9)	3996 (11.7)	0.01
Hemorrhagic stroke	655 (1.9)	649 (1.9)	0
Hypertension	18 331 (53.5)	18 313 (53.5)	0
Inflammatory bowel disease	88 (0.3)	82 (0.2)	0
Ischemic heart disease	6426 (18.8)	6328 (18.5)	0.01
Ischemic stroke	2242 (6.6)	2217 (6.5)	0
Macular degeneration	1766 (5.2)	1750 (5.1)	0
Multiple sclerosis	17 (0.1)	16 (0.1)	0
Osteoarthritis	17 098 (49.9)	17 033 (49.7)	0
Parkinson disease	1830 (5.3)	1790 (5.2)	0.01
Peptic ulcer	8450 (24.7)	8378 (24.5)	0
Pneumonia	3317 (9.7)	3314 (9.7)	0
Kidney failure	3664 (10.7)	3624 (10.6)	0
Rheumatoid arthritis	1873 (5.5)	1931 (5.6)	−0.01
Sarcoidosis	8 (0.0)	11 (0.0)	−0.01
Schizophrenia	144 (0.4)	148 (0.4)	−0.00
Systemic lupus erythematosus	344 (1.0)	334 (1.0)	0
Previous osteoporosis medication			
Oral or intravenous bisphosphonate	8395 (24.5)	8346 (24.4)	0
Teriparatide	1547 (4.5)	1558 (4.6)	0
Calcitonin	1161 (3.4)	1127 (3.3)	0.01
Raloxifene	2847 (8.3)	2824 (8.2)	0
Baseline medication			
α-Blocker	1071 (3.1)	1080 (3.2)	0
Antacid	19 895 (58.1)	19 772 (57.7)	0.01
Antiarrhythmic	3069 (9.0)	3076 (9.0)	0
Antidementia	2987 (8.7)	2904 (8.5)	0.01
Antidepressant	7043 (20.6)	6952 (20.3)	0.01
Antigout	3377 (9.9)	3293 (9.6)	0.01
Antihistamine	22907 (66.9)	22895 (66.8)	0
Anti-Parkinson	2900 (8.5)	2861 (8.4)	0
Antiplatelet	10 941 (31.9)	10 891 (31.8)	0
Antipsychotic	6135 (17.9)	6078 (17.7)	0
Antithrombotic	690 (2.0)	663 (1.9)	0.01
Benzodiazepine	22 494 (65.7)	22 414 (65.4)	0
β-blocker	10 965 (32.0)	10 840 (31.7)	0.01
Bronchodilator	15030 (43.9)	14882 (43.4)	0.01
Calcium channel blocker	15233 (44.5)	15245 (44.5)	0
Diuretic	9442 (27.6)	9314 (27.2)	0.01
Hormone replacement therapy	780 (2.3)	734 (2.1)	0.01
Lipid lowering agent	7940 (23.2)	7917 (23.1)	0
NSAID	30 940 (90.3)	30 904 (90.2)	0
Propulsive agent	16 743 (48.9)	16 499 (48.2)	0.01
Quetiapine	3058 (8.9)	2991 (8.7)	0.01
RAS-acting agent	14 060 (41.1)	14 070 (41.1)	0

Abbreviations: COPD, chronic obstructive pulmonary disease; NSAID, nonsteroidal anti-inflammatory drug; NT$, Taiwanese new dollar; RAS, renin-angiotensin system.

^a^ The treatment group included patients who continued to receive denosumab; patients in the comparison group discontinued the drug.

### Statistical Analysis

Propensity score matching was performed to balance the baseline differences and to control for potential confounding factors. A propensity score was initially calculated for each patient to estimate the probability of being assigned to the treatment group by using multivariable logistic regression models based on all the covariates listed in Table 1. The propensity score was then used to match the treatment and comparison groups in a 1:1 ratio using the greedy 8→1 digit algorithm.^24^ For the matched study population, we used the Kaplan-Meier method to estimate the cumulative incidence curve and a Cox proportional hazards model to estimate the hazard ratio (HR). The proportional hazards assumption was examined using the supremum tests, and no significant violation was found (*P* = .17).^25^ The statistical significance level was set at a 2-tailed *P* < .05 for all tests. Data management and statistical analyses were performed using SAS software, version 9.4 (SAS Institute Inc). Data were analyzed from January 1 to November 30, 2023.

Several subgroup analyses were performed, including those stratified by age (<65 and ≥65 years); sex; and presence of dyslipidemia, hypertension, ischemic heart disease, and kidney failure. We performed various sensitivity analyses to determine the robustness of our findings. First, we performed a sensitivity analysis using an as-started design (analogue of the intention-to-treat design); the follow-up did not censor when denosumab was discontinued in the treatment group or when the osteoporosis treatment was started or restarted in the comparison group. Second, we conducted a sensitivity analysis that adjusted for the use of other antiosteoporosis medications (eg, bisphosphonates, teriparatide, raloxifene, and calcitonin) during the follow-up as time-varying covariates in the Cox regression models. Third, we used another propensity score method, stabilized inverse probability of treatment weighting (IPTW), to control for confounders in the sensitivity analysis.^26^ Fourth, we included all eligible patients without applying propensity score methods and used multivariable Cox proportional hazards models with adjustments for all the covariates listed in Table 1 to obtain the adjusted HR. Fifth, a sensitivity analysis applying Fine-Gray subdistribution hazards models, considering death as a competing risk, was performed.^27^ Moreover, we examined several negative control outcomes (ie, diagnoses of asthma, lung cancer, and skin cancer, as well as the use of antidepressants or thiazides) that were not expected to have any causal link to denosumab. This indirect assessment explored the existence of potential unmeasured confounding.^28^

## Results

### Patient Characteristics

In total, 101 296 patients who had received an initial dose of denosumab were recruited for the study population (66 399 in the treatment group and 34 897 in the comparison group). The mean (SD) age was 76.6 (9.8) years; 85.9% of the population was female. Most patient characteristics were similar between the 2 groups (eTable 2 in Supplement 1). After propensity score matching, 68 510 patients were included (mean [SD] age, 77.7 [9.8] years; 57 762 [84.3%] women and 10 748 [15.7%] men); 34 255 individuals in the treatment group were matched with 34 255 individuals in the comparison group. All baseline characteristics were well balanced between the treatment and comparison groups, with standardized differences of all covariates being below 0.10 (Table 1). The patient selection flowchart is shown in eFigure 2 in Supplement 1.

### Risk of Developing Diabetes

**Figure 1.**
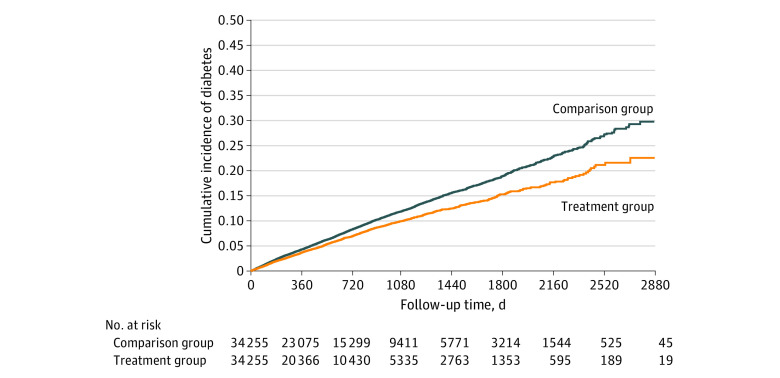
Cumulative Incidence Curves of Diabetes for the Treatment and Comparison Groups The treatment group included patients who continued to receive denosumab; patients in the comparison group discontinued the drug.

During a mean (SD) follow-up of 1.9 (1.6) years, 2016 patients developed diabetes in the treatment group, and 3220 patients developed diabetes in the comparison group (incidence rates, 35.9 vs 43.6 per 1000 person-years). The Kaplan-Meier curves suggested a lower cumulative incidence of diabetes in the denosumab treatment group than in the comparison group (Figure 1). Cox proportional hazards models indicated that denosumab treatment was associated with lower risk of incident diabetes (HR, 0.84; 95% CI, 0.78-0.90) (Table 2).

**Table 2.** Risk of Diabetes Development in the Treatment and Comparison Groups^a^

Metric	Group	
	Treatment (n = 34 255)	Comparison (n = 34 255)
Diabetes event, No.	2016	3220
Person-years	56 184.6	73 884.9
Incidence rate^b^	35.9	43.6
HR (95% CI)^c^	0.84 (0.78-0.90)	1 [Reference]

Abbreviation: HR, hazard ratio.

^a^ The treatment group included patients who continued to receive denosumab; patients in the comparison group discontinued the drug.

^b^ Per 1000 person-years.

^c^ Calculated using the Cox proportional hazards model in the propensity score–matched population.

### Stratified Analyses

**Figure 2.**
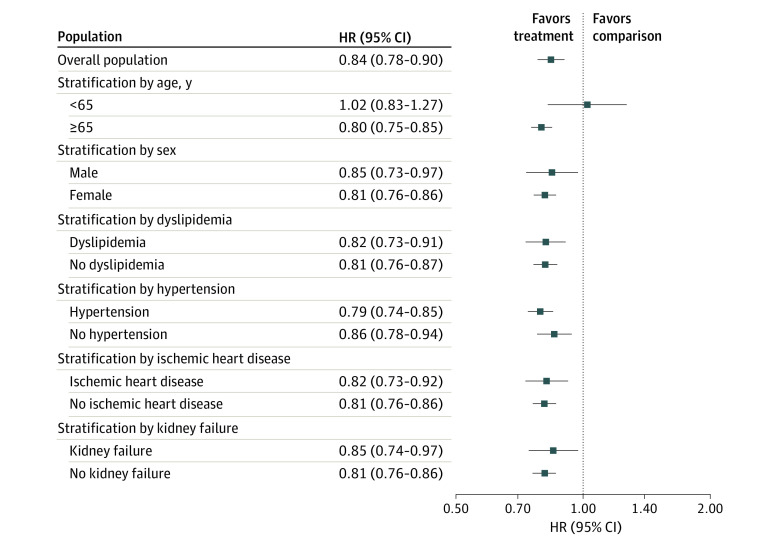
Comparison of Diabetes Risk Between Treatment and Comparison Group The treatment group included patients who continued to receive denosumab; patients in the comparison group discontinued the drug.

Among patients 65 years or older, the risk of diabetes was significantly lower in the denosumab treatment group than in the comparison group (HR, 0.80; 95% CI, 0.75-0.85); however, this difference was not observed in patients younger than 65 years (HR, 1.02; 95% CI, 0.83-1.27). A lower risk of diabetes associated with denosumab treatment was found in both male (HR, 0.85; 95% CI, 0.73-0.97) and female (HR, 0.81; 95% CI, 0.76-0.86) subgroups. Lower diabetes risk with denosumab treatment was also observed regardless of whether patients had the following comorbidities: dyslipidemia (with dyslipidemia: HR, 0.82 [95% CI, 0.73-0.91]; without dyslipidemia: HR, 0.81 [95% CI, 0.76-0.87]), hypertension (with hypertension: HR, 0.79 [95% CI, 0.74-0.85]; without hypertension: HR, 0.86 [95% CI, 0.78-0.94]), ischemic heart disease (with ischemic heart disease: HR, 0.82 [95% CI, 0.73-0.92]; without ischemic heart disease: HR, 0.81 [95% CI, 0.76-0.86]), or kidney failure (with kidney failure: HR, 0.85 [95% CI, 0.74-0.97]; without kidney failure: HR, 0.81 [95% CI, 0.76-0.86]) (Figure 2; eTable 3 in Supplement 1).

### Sensitivity Analyses

The sensitivity analysis applying an as-started design (analogue of the intention-to-treat design) demonstrated that denosumab treatment was associated with lower risk of diabetes development (HR, 0.91; 95% CI, 0.87-0.96). A sensitivity analysis that adjusted for using other antiosteoporosis medications during the follow-up as time-varying covariates also showed similar results (HR, 0.82; 95% CI, 0.77-0.86). The sensitivity analyses applying stabilized IPTW (HR, 0.79; 95% CI, 0.75-0.83) and using only multivariable regression models (HR, 0.80; 95% CI, 0.76-0.84) to control for potential confounders also demonstrated findings consistent with our primary analyses. The sensitivity analysis considering death as a competing risk also showed similar results (HR, 0.83; 95% CI, 0.79-0.88) (Table 3). Regarding the analyses of negative control outcomes, none of those outcomes significantly differed between the denosumab treatment and comparison groups (eTable 4 in Supplement 1).

**Table 3.** Sensitivity Analyses to Evaluate Risk of Diabetes Development in the Treatment and Comparison Groups^a^

Analysis	Participants, No.	Event, No.	Person-years	Incidence rate^b^	HR (95% CI)
As-started design^c^					
Treatment group	34 255	3657	93 493.2	39.1	0.91 (0.87-0.96)
Comparison group	34 255	3894	90 844.1	42.9	1 [Reference]
Cox models with time-varying covariates^d^					
Treatment group	34 255	2016	56 184.6	35.9	0.82 (0.77-0.86)
Comparison group	34 255	3894	90 844.1	42.9	1 [Reference]
Stabilized IPTW^e^					
Treatment group	66 399	3688.7	111 343.8	33.1	0.79 (0.75-0.83)
Comparison group	34 959	3138.3	75 601.2	41.5	1 [Reference]
Multivariable regression models without propensity score methods^f^					
Treatment group	66 399	3584	11 2944.7	31.7	0.80 (0.76-0.84)
Comparison group	34 897	3337	74 929.7	44.5	1 [Reference]
Death as a competing risk^g^					
Treatment group	34 255	2016	60 597.1	33.27	0.83 (0.79-0.88)
Comparison group	34 255	3220	82 963.9	38.81	1 [Reference]

Abbreviations: HR, hazard ratio; IPTW, inverse probability of treatment weighting.

^a^ The treatment group included patients who continued to receive denosumab; patients in the comparison group discontinued the drug.

^b^ Per 1000 person-years.

^c^ The follow-up did not censor when denosumab was discontinued in the treatment group or when the osteoporosis treatment was started or restarted in the comparison group (analogue of intention-to-treat design).

^d^ Adjusted for the use of other antiosteoporosis medications (eg, bisphosphonates, teriparatide, raloxifene, and calcitonin) during follow-up as time-varying covariates in Cox regression models.

^e^ Using stabilized IPTW instead of propensity score matching to control for potential confounding effects.

^f^ Including all eligible patients without applying propensity score methods and using multivariable Cox proportional hazards models for confounder adjustment.

^g^ Applying Fine-Gray subdistribution hazards models considering death as a competing risk.

## Discussion

In this large-scale, nationwide cohort study, patients with continued treatment of denosumab for osteoporosis had significantly lower risk of developing diabetes than patients with discontinued denosumab treatment, suggesting a potential association of denosumab therapy with lowering diabetes risk. These results were supported by several sensitivity analyses, confirming the robustness of the findings. The age-stratified analyses further suggested that the decreased risk of diabetes associated with continued denosumab treatment was observed specifically among adults 65 years or older.

To date, clinical evidence on the effect of denosumab on diabetes risk is limited and inconsistent. A post hoc analysis of the FREEDOM trial indicated no significant effect of denosumab on reducing diabetes risk (relative risk, 0.85; 95% CI, 0.61-1.17),^14^ with potential underpowering hindering conclusive findings. In contrast, a recent retrospective cohort study in the UK suggested reduced risk of diabetes associated with denosumab use compared with bisphosphonate use (HR, 0.68; 95% CI, 0.52-0.89).^15^ However, confounding by indication may not have been completely eliminated in that study because factors such as kidney function and upper gastrointestinal tract diseases may be associated with the selection of denosumab or bisphosphonates. Moreover, the potential association of bisphosphonates with reduced diabetes risk, as suggested by previous studies,^29,30^ may influence the comparisons between bisphosphonates and denosumab and complicate the use of bisphosphonates as an appropriate reference group.

To address these concerns, we conducted a large-scale, real-world (clinical practice) cohort study to evaluate whether the continuation of denosumab treatment, in contrast to its discontinuation, modifies the risk of developing diabetes. As a single dose of denosumab is not expected to offer a sustained clinical advantage,^21,31^ this approach helped to simulate the placebo group in a clinical trial. Several extensive sensitivity analyses were performed, all of which supported the main results of our study. Therefore, this study provides additional valuable and reliable evidence that denosumab treatment is associated with lower risk of incident diabetes, helping to fill the current knowledge gap. Notably, we found a different discontinuation rate of denosumab treatment between our study and a previous study conducted in the UK by Lyu et al.^15^ The plausible reasons for this difference could include differences in study designs, database duration, race and ethnicity, health care systems, and cultural factors.

Based on the incidence rates for the treatment and comparison groups (35.9 vs 43.6 per 1000 person-years), the estimated number of patients needed to treat to prevent 1 additional diabetes case was approximately 130 per year. Previous research has shown no significant difference in the overall risk of adverse events between denosumab and other common antiosteoporotic drugs, such as bisphosphonates.^32^ Therefore, in choosing osteoporosis treatments, the potential for increased adverse event risks associated with denosumab may not be a primary concern.^33,34^ Given the high osteoporosis prevalence, the extensive use of antiosteoporosis medications, and the negative effect of diabetes on both patient health and health care system burdens in the global aging population, our findings possess substantial clinical and public health significance.

In the age-stratified analysis, a significantly lower risk of diabetes with denosumab treatment was found specifically for patients 65 years or older. Given that the prevalence and incidence of diabetes are higher in older adults than in younger ones,^35^ a finding of an association between denosumab treatment and lower diabetes risk in older adults but no such association in younger adults was reasonable. This result suggests that our findings should be particularly considered in this vulnerable population.

The present study could not determine the underlying biological mechanisms of the association between denosumab and a lower risk of diabetes; however, some previous preclinical studies may help explain these findings. A close correlation between receptor activator of nuclear factor κB ligand (RANKL) inhibition and improved glucose metabolism has previously been proposed.^11^ Low-grade inflammation is associated with the onset of insulin resistance and diabetes. Receptor activator of nuclear factor κB ligand is a potent activator of nuclear factor κB, which is a primary proinflammatory switch that can modulate inflammation levels. It has been hypothesized that systemic and hepatic insulin resistance could result from subacute inflammation caused by minor activation of nuclear factor κB. Hence, mitigating RANKL using denosumab may reduce subacute inflammation and improve insulin resistance.^11,36^ Another hypothesized mechanism is that RANKL suppression may promote β-cell proliferation.^37^ The failure of β cells is a fundamental pathogenic process in diabetes.^38^ Since the RANKL/RANK pathway inhibits β-cell replication in humans, suppressing the RANKL/RANK pathway via denosumab may enhance human β-cell replication.^37^ Such compelling evidence suggests potential underlying mechanisms for the association between denosumab and preventing diabetes development.

### Limitations

This study has some limitations. First, due to the inherent limitations of using claims-based data in research, we could not obtain some clinical details, such as lifestyle, substance use, prediabetes, weight status, and laboratory results (eg, exact blood glucose levels and lipid profiles). The exact reasons for discontinuing treatment in the comparison group were uncertain. Because Taiwan’s National Health Insurance covered the use of denosumab without copayment, we considered the financial burden of the drugs not to be the major reason for the discontinuation. However, socioeconomic factors may also affect patients’ behavior; thus, we considered indicators such as income level, urban level of residence, location of residence, and health care use to address the issues. Although we used propensity score matching methods to control for many covariates, residual confounding factors could not be ruled out completely. The association of denosumab with lowering diabetes risk may be overestimated when residual confounders related to socioeconomic status are not controlled entirely. Second, differential censoring was inevitable because the allocation to treatment and comparison groups was based on the decision to either continue or discontinue denosumab treatment. The specific reasons for censoring in each group are detailed in eTable 5 in Supplement 1. Nevertheless, our sensitivity analysis, which applied an as-started design (comparable to intention-to-treat design) that did not censor patients who changed or stopped use of antiosteoporosis drugs, also yielded findings consistent with our primary analysis. The sensitivity analysis considering death as a competing risk also showed similar results. These results further support the robustness of our study findings. Third, because of the anonymity policy of the NHIRD, we could not directly evaluate patients to validate incident diabetes. However, the development of diabetes in this study was confirmed only when patients received antidiabetic treatment; this approach may help ascertain a high positive predictive value of diabetes diagnosis. Even if underreporting of diabetes or misclassification errors remained possible, such situations would be expected to occur nondifferentially among the study groups, which would bias the estimates toward the null.^39,40^ As our results showed a significant association between denosumab treatment and a lower diabetes risk, the exact association between denosumab therapy and lowering diabetes risk may be greater than that observed. Fourth, even though our study results were based on analyzing almost the entire Taiwanese population, whether our findings could be extrapolated to other races and ethnicities or countries remains undetermined and needs further exploration.

## Conclusions

The findings of this cohort study indicated that denosumab treatment was associated with lower risk of incident diabetes. The age-stratified analyses further indicated a lower diabetes risk with denosumab treatment specifically in adults 65 years or older. These findings may help physicians choose an appropriate antiosteoporosis medication for patients with osteoporosis while also considering a medication associated with lowering diabetes risk. This study provides a fundamental backdrop for future prospective studies or randomized clinical trials to validate the findings on denosumab use and its association with reduced diabetes risk.

## Notes

Supplement 1. **eAppendix 1.** The specific datasets from the National Health Insurance Research Database (NHIRD) that we used for data analyses **eFigure 1.** Schema of the study design **eFigure 2.** Flowchart of patient selection **eTable 1.** Diagnosis and drug codes for identifying baseline comorbidities, co-medications, and negative control outcomes **eTable 2.** Baseline characteristics of the denosumab treatment and comparison groups in the original population without propensity score matching **eTable 3.** Detailed results of the analyses stratified by age, sex, and comorbidities **eTable 4.** Results of the negative control outcome analyses **eTable 5.** Reasons for end of follow-up or censoring in the treatment and comparison groups in the primary analysis

Supplement 2.
Data Sharing Statement

## References

[1] Clynes MA, Harvey NC, Curtis EM, Fuggle NR, Dennison EM, Cooper C. The epidemiology of osteoporosis. *Br Med Bull.* 2020;133(1):105-117. doi:10.1093/bmb/ldaa005 PMC7115830 32282039

[2] NIH Consensus Development Panel on Osteoporosis Prevention, Diagnosis, and Therapy. Osteoporosis prevention, diagnosis, and therapy. *JAMA*. 2001;285(6):785-795. doi:10.1001/jama.285.6.785 11176917

[3] Johnston CB, Dagar M. Osteoporosis in older adults. Med Clin North Am. 2020;104(5):873-884. doi:10.1016/j.mcna.2020.06.004 32773051

[4] Hofbauer LC, Busse B, Eastell R, . Bone fragility in diabetes: novel concepts and clinical implications. Lancet Diabetes Endocrinol. 2022;10(3):207-220. doi:10.1016/S2213-8587(21)00347-8 35101185

[5] Hsu JY, Cheng CY, Hsu CY. Type 2 diabetes mellitus severity correlates with risk of hip fracture in patients with osteoporosis. Neth J Med. 2018;76(2):65-71. 29515003

[6] Lui DTW, Lee CH, Chan YH, . HbA1c variability, in addition to mean HbA1c, predicts incident hip fractures in Chinese people with type 2 diabetes. Osteoporos Int. 2020;31(10):1955-1964. doi:10.1007/s00198-020-05395-z 32385660

[7] Li CI, Liu CS, Lin WY, . Glycated hemoglobin level and risk of hip fracture in older people with type 2 diabetes: a competing risk analysis of Taiwan diabetes cohort study. J Bone Miner Res. 2015;30(7):1338-1346. doi:10.1002/jbmr.2462 25598134

[8] Cipriani C, Colangelo L, Santori R, . The interplay between bone and glucose metabolism. Front Endocrinol (Lausanne). 2020;11:122. doi:10.3389/fendo.2020.00122 32265831 PMC7105593

[9] Paschou SA, Dede AD, Anagnostis PG, Vryonidou A, Morganstein D, Goulis DG. Type 2 diabetes and osteoporosis: a guide to optimal management. J Clin Endocrinol Metab. 2017;102(10):3621-3634. doi:10.1210/jc.2017-00042 28938433

[10] Dede AD, Tournis S, Dontas I, Trovas G. Type 2 diabetes mellitus and fracture risk. Metabolism. 2014;63(12):1480-1490. doi:10.1016/j.metabol.2014.09.002 25284729

[11] Kiechl S, Wittmann J, Giaccari A, . Blockade of receptor activator of nuclear factor-κB (RANKL) signaling improves hepatic insulin resistance and prevents development of diabetes mellitus. Nat Med. 2013;19(3):358-363. doi:10.1038/nm.3084 23396210

[12] Napoli N, Pannacciulli N, Vittinghoff E, . Effect of denosumab on fasting glucose in women with diabetes or prediabetes from the FREEDOM trial. Diabetes Metab Res Rev. 2018;34(4):e2991. doi:10.1002/dmrr.2991 29430796

[13] Pacheco-Soto BT, Elguezabal-Rodelo RG, Porchia LM, Torres-Rasgado E, Pérez-Fuentes R, Gonzalez-Mejia ME. Denosumab improves glucose parameters in patients with impaired glucose tolerance: a systematic review and meta-analysis. J Drug Assess. 2021;10(1):97-105. doi:10.1080/21556660.2021.1989194 34676131 PMC8525927

[14] Schwartz AV, Schafer AL, Grey A, . Effects of antiresorptive therapies on glucose metabolism: results from the FIT, HORIZON-PFT, and FREEDOM trials. *J Bone Miner Res**.* 2013;28(6):1348-1354. doi:10.1002/jbmr.186 23322676

[15] Lyu H, Zhao SS, Zhang L, . Denosumab and incidence of type 2 diabetes among adults with osteoporosis: population based cohort study. BMJ. 2023;381:e073435. doi:10.1136/bmj-2022-073435 37072150 PMC10111187

[16] von Elm E, Altman DG, Egger M, Pocock SJ, Gøtzsche PC, Vandenbroucke JP; STROBE Initiative. The Strengthening the Reporting of Observational Studies in Epidemiology (STROBE) statement: guidelines for reporting observational studies. PLoS Med. 2007;4(10):e296. doi:10.1371/journal.pmed.0040296 17941714 PMC2020495

[17] Benchimol EI, Smeeth L, Guttmann A, ; RECORD Working Committee. The REporting of studies Conducted using Observational Routinely-collected health Data (RECORD) statement. PLoS Med. 2015;12(10):e1001885. doi:10.1371/journal.pmed.1001885 26440803 PMC4595218

[18] Hsieh CY, Su CC, Shao SC, . Taiwan’s National Health Insurance Research Database: past and future. Clin Epidemiol. 2019;11:349-358. doi:10.2147/CLEP.S196293 31118821 PMC6509937

[19] Hsing AW, Ioannidis JP. Nationwide population science: lessons from the Taiwan National Health Insurance Research Database. JAMA Intern Med. 2015;175(9):1527-1529. doi:10.1001/jamainternmed.2015.3540 26192815

[20] Tai TW, Huang CF, Huang HK, . Clinical practice guidelines for the prevention and treatment of osteoporosis in Taiwan: 2022 update. J Formos Med Assoc. 2023;122(suppl 1):S4-S13. doi:10.1016/j.jfma.2023.01.007 36781371

[21] Cummings SR, San Martin J, McClung MR, ; FREEDOM Trial. Denosumab for prevention of fractures in postmenopausal women with osteoporosis. N Engl J Med. 2009;361(8):756-765. doi:10.1056/NEJMoa0809493 19671655

[22] Lévesque LE, Hanley JA, Kezouh A, Suissa S. Problem of immortal time bias in cohort studies: example using statins for preventing progression of diabetes. BMJ. 2010;340:b5087. doi:10.1136/bmj.b5087 20228141

[23] Dafni U. Landmark analysis at the 25-year landmark point. Circ Cardiovasc Qual Outcomes. 2011;4(3):363-371. doi:10.1161/CIRCOUTCOMES.110.957951 21586725

[24] Parsons LS; Ovation Research Group. Reducing bias in a propensity score matched-pair sample using greedy matching techniques. Proceedings of the Twenty-Sixth Annual SAS Users Group International Conference, Long Beach, CA; 2001. Accessed June 20, 2022. https://support.sas.com/resources/papers/proceedings/proceedings/sugi26/p214-26.pdf

[25] Lin DY, Wei LJ, Ying Z. Checking the Cox model with cumulative sums of martingale-based residuals. Biometrika. 1993;80(3):557-572. doi:10.1093/biomet/80.3.557

[26] Desai RJ, Franklin JM. Alternative approaches for confounding adjustment in observational studies using weighting based on the propensity score: a primer for practitioners. BMJ. 2019;367:l5657. doi:10.1136/bmj.l5657 31645336

[27] Fine JP, Gray RJ. A proportional hazards model for the subdistribution of a competing risk. J Am Stat Assoc. 1999;94(446):496-509. doi:10.1080/01621459.1999.10474144

[28] Lipsitch M, Tchetgen Tchetgen E, Cohen T. Negative controls: a tool for detecting confounding and bias in observational studies. Epidemiology. 2010;21(3):383-388. doi:10.1097/EDE.0b013e3181d61eeb 20335814 PMC3053408

[29] Anastasilakis AD, Tsourdi E, Tabacco G, . The impact of antiosteoporotic drugs on glucose metabolism and fracture risk in diabetes: good or bad news? J Clin Med. 2021;10(5):996. doi:10.3390/jcm10050996 33801212 PMC7957889

[30] Chen PW, Su HY, Tu YK, . Association of bisphosphonates with diabetes risk and glycemic control: a meta-analysis. Osteoporos Int. 2023;34(2):387-397. doi:10.1007/s00198-022-06616-3 36464699

[31] Lai EC, Lin TC, Lange JL, . Effectiveness of denosumab for fracture prevention in real-world postmenopausal women with osteoporosis: a retrospective cohort study. *Osteoporos Int*. 2022;33(5):1155-1164. doi:10.1007/s00198-021-06291-w PMC9007768 35032187

[32] Wu J, Zhang Q, Yan G, Jin X. Denosumab compared to bisphosphonates to treat postmenopausal osteoporosis: a meta-analysis. J Orthop Surg Res. 2018;13(1):194. doi:10.1186/s13018-018-0865-3 30071889 PMC6090940

[33] Chen Y, Zhu J, Zhou Y, Peng J, Wang B. Efficacy and Safety of Denosumab in Osteoporosis or Low Bone Mineral Density Postmenopausal Women. Front Pharmacol. 2021;12:588095. doi:10.3389/fphar.2021.588095 33935694 PMC8080120

[34] Rhee Y, Chang DG, Ha J, . Real-World Safety and Effectiveness of Denosumab in Patients with Osteoporosis: A Prospective, Observational Study in South Korea. Endocrinol Metab (Seoul). 2022;37(3):497-505. doi:10.3803/EnM.2022.1427 35654577 PMC9262695

[35] Kirkman MS, Briscoe VJ, Clark N, . Diabetes in older adults. Diabetes Care. 2012;35(12):2650-2664. doi:10.2337/dc12-1801 23100048 PMC3507610

[36] Cai D, Yuan M, Frantz DF, . Local and systemic insulin resistance resulting from hepatic activation of IKK-beta and NF-kappaB. Nat Med. 2005;11(2):183-190. doi:10.1038/nm1166 15685173 PMC1440292

[37] Kondegowda NG, Fenutria R, Pollack IR, . Osteoprotegerin and Denosumab Stimulate Human Beta Cell Proliferation through Inhibition of the Receptor Activator of NF-κB Ligand Pathway. Cell Metab. 2015;22(1):77-85. doi:10.1016/j.cmet.2015.05.021 26094891 PMC4597781

[38] Eizirik DL, Pasquali L, Cnop M. Pancreatic β-cells in type 1 and type 2 diabetes mellitus: different pathways to failure. Nat Rev Endocrinol. 2020;16(7):349-362. doi:10.1038/s41574-020-0355-7 32398822

[39] Copeland KT, Checkoway H, McMichael AJ, Holbrook RH. Bias due to misclassification in the estimation of relative risk. Am J Epidemiol. 1977;105(5):488-495. doi:10.1093/oxfordjournals.aje.a112408 871121

[40] Höfler M. The effect of misclassification on the estimation of association: a review. Int J Methods Psychiatr Res. 2005;14(2):92-101. doi:10.1002/mpr.20 16175878 PMC6878572

