# Supplementary material for: Denosumab and the Risk of Diabetes in Patients Treated for Osteoporosis
Source: JAMA Netw Open. 2024 Feb 9;7(2):e2354734. doi: 10.1001/jamanetworkopen.2023.54734 (PMC10858399; doi:10.1001/jamanetworkopen.2023.54734)
Supplement: Supplement 1. — eAppendix 1. The specific datasets from the National Health Insurance Research Database (NHIRD) that we used for data analyses eFigure 1. Schema of the study design eFigure 2. Flowchart of patient selection eTable 1. Diagnosis and drug codes for identifying baseline comorbidities, co-medications, and negative control outcomes eTable 2. Baseline characteristics of the denosumab treatment and comparison groups in the original population without propensity score matching eTable 3. Detailed results of the analyses stratified by age, sex, and comorbidities eTable 4. Results of the negative control outcome analyses eTable 5. Reasons for end of follow-up or censoring in the treatment and comparison groups in the primary analysis [file jamanetwopen-e2354734-s001.pdf]

# Supplementary Online Content

Huang HK, Chuang ATM, Liao TC, et al. Denosumab and the risk of diabetes in patients treated for osteoporosis. *JAMA Netw Open*. 2024;7(2):e2354734. doi:10.1001/jamanetworkopen.2023.54734

**eAppendix 1.** The specific datasets from the National Health Insurance Research Database (NHIRD) that we used for data analyses
**eReferences.**
**eFigure 1.** Schema of the study design
**eFigure 2.** Flowchart of patient selection
**eTable 1.** Diagnosis and drug codes for identifying baseline comorbidities, co-medications, and negative control outcomes
**eTable 2.** Baseline characteristics of the denosumab treatment and comparison groups in the original population without propensity score matching
**eTable 3.** Detailed results of the analyses stratified by age, sex, and comorbidities
**eTable 4.** Results of the negative control outcome analyses
**eTable 5.** Reasons for end of follow-up or censoring in the treatment and comparison groups in the primary analysis

This supplementary material has been provided by the authors to give readers additional information about their work.

© 2024 Huang HK et al. *JAMA Network Open*.

**eAppendix 1.** The specific datasets from the National Health Insurance Research Database(NHIRD) that we used for data analyses

The demographic data, medical claims for inpatient, outpatient, and emergency care services, and drug prescription details that we used for analyses were sourced from several datasets within Taiwan's NHIRD. These datasets include Registry for Beneficiaries, Ambulatory Care Expenditures by Visits, Inpatient Expenditures by Admissions, Expenditures for Prescriptions Dispensed at Contracted Pharmacies, Details of Ambulatory Care Orders, Details of Inpatient Orders, and Details of Prescriptions Dispensed at Contracted Pharmacies. The NHIRD covers prescription data, dispensing data, and all claims data. Mortality information was obtained by cross-referencing the NHIRD with the Taiwan National Register of Death. The NHIRD is maintained by the Health and Welfare Data Science Center, Ministry of Health and Welfare in Taiwan, and is accessible for formal research applications. For more detailed information on the NHIRD, please refer to the following references.[1,2]

## eReferences.


1. Hsieh CY, Su CC, Shao SC, et al. Taiwan's National Health Insurance Research Database: past and future. *Clin Epidemiol*. 2019;11:349-58.
2. Hsing AW, Ioannidis JP. Nationwide population science: lessons from the Taiwan National Health Insurance Research Database. *JAMA Intern Med*. 2015;175(9):1527-9.


© 2024 Huang HK et al. *JAMA Network Open*.

**eFigure 1.** Schema of the study design

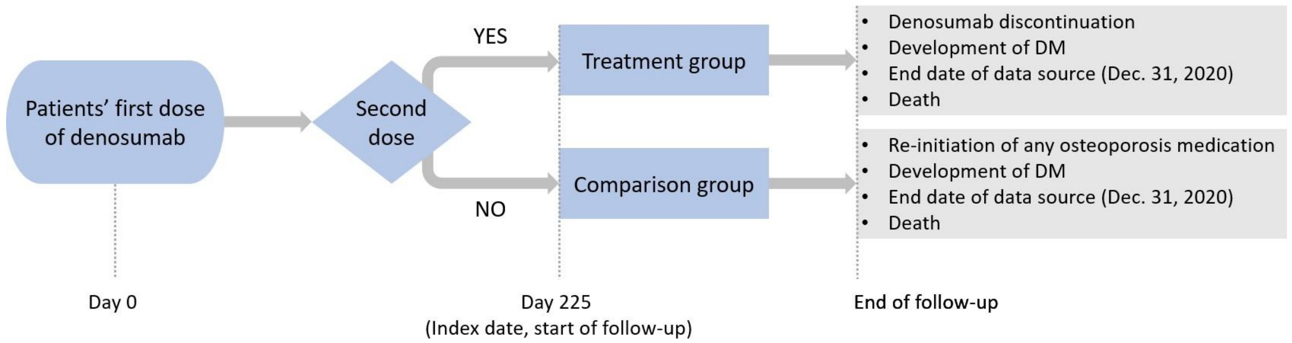


© 2024 Huang HK et al. *JAMA Network Open*.

**eFigure 2.** Flowchart of patient selection

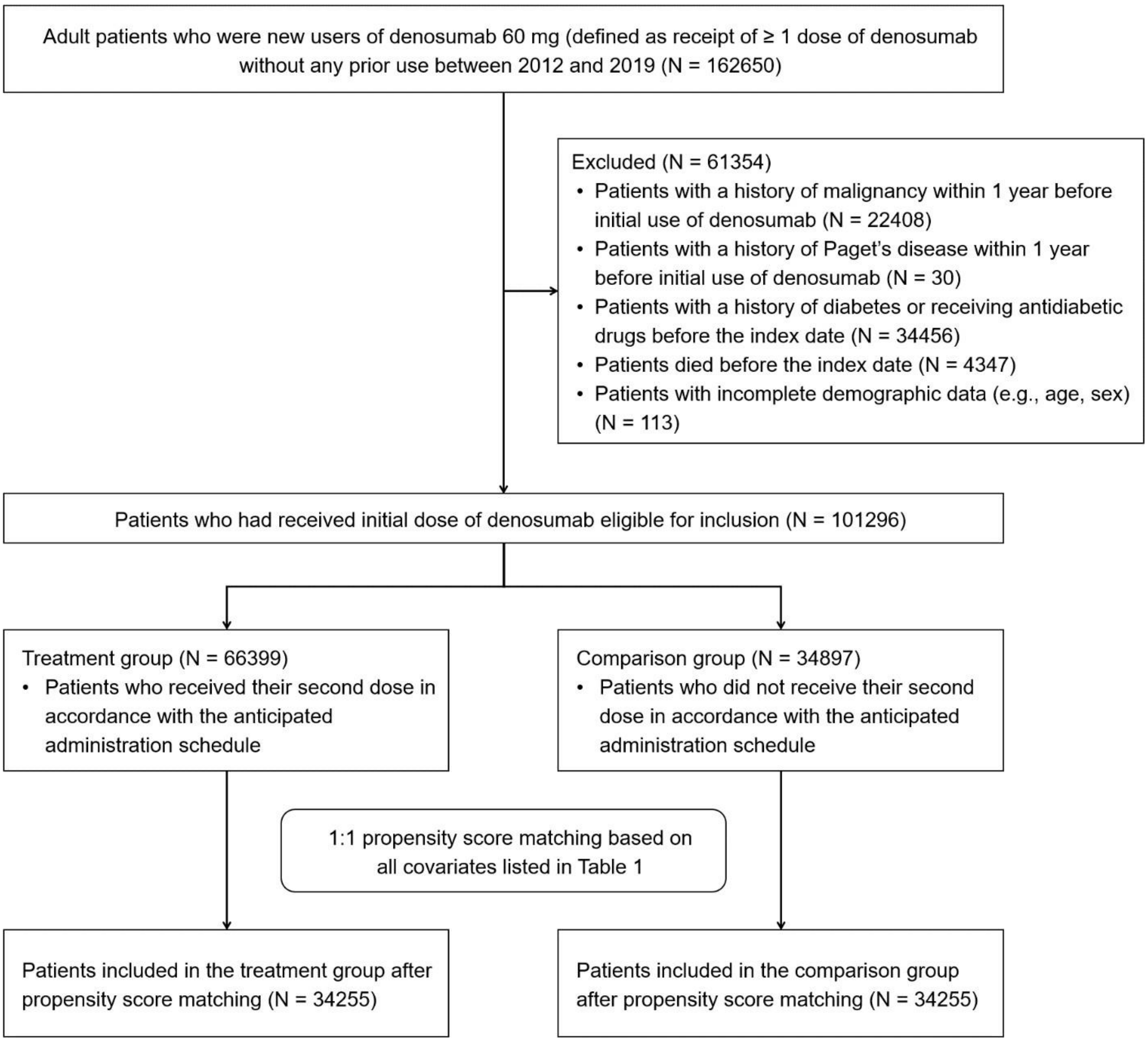


© 2024 Huang HK et al. *JAMA Network Open*.

**eTable 1**. Diagnosis and drug codes for identifying baseline comorbidities, co-medications, and negative control outcomes

| **Comorbidities** | **ICD-9-CM / ICD-10-CM codes** |
|---|---|
| Chronic Obstructive Pulmonary Disease | 491, 492, J41, J42, J44 |
| Systemic Lupus Erythematosus | 7100, M32 |
| Asthma | 493, J45 |
| Inflammatory bowel disease | 556, K51 |
| Multiple sclerosis | 340, G35 |
| Sarcoidosis | 135, D86 |
| Gout | 274, M10 |
| Rheumatoid arthritis | 714, M05, M06 |
| Cataract | 366, H25 |
| Congestive heart failure | 428, I50 |
| Dementia | 290, F01, F03, F05 |
| Depression | 311, F32, F33 |
| Diabetes mellitus | 250, E10, E11 |
| Dyslipidemia | E780, E781, E782, E783, E784, E785, 272 |
| Gastrointestinal bleeding | 578, K920, K921, K922 |
| Glaucoma | 365, H40, H42 |
| Hemorrhagic stroke | 430, 431, 432, I60, I61, I62 |
| Hypertension | 401, 402, 403, 404, 405, I10 |
| Ischemic heart disease | 410, 411, 412, 413, 414, I20, I21, I22, I23, I24, I25 |
| Ischemic stroke | 433, 434, I63 |
| Lung cancer | 162, C33, C34 |
| Macular degeneration | 3625, H353 |
| Osteoarthritis | 715, M15, M16, M17, M18, M19 |
| Parkinson disease | 332, G20 |
| Peptic ulcer | 531-534 or K25, K26, K27, K28 |
| Pneumonia | J12, J13, J14, J15, J16, J17, J18, 480-486 |
| Renal failure | 584, 585, 586, N18 |
| Schizophrenia | 295, F20 |
| Skin cancer | 172, 173, C43, C44, C4A |
| Hip fracture | 820, 820.2, 820.8, 733.14, S72.0--A, S72.1--A, S72.2--A, M80.051A, M80.052A, M80.059A, M84.451A, M84.452A, |
| Vertebral fracture | 805.0, 805.2, 805.4, 805.8, 733.13, S12.---A, S22.0--A, S32.0--A , M48.50XA, M48.52XA, M48.53XA |
| Humerus fracture | 812, 812.2, 812.4, 733.11, S42.2--A, S42.3--A, S42.4--A, M80.021A, M80.022A, M80.029A, M84.421A, M84.422A, M84.429A |

© 2024 Huang HK et al. *JAMA Network Open*.

| | |
|---|---|
| Wrist fracture | 813.4, 813.5,733.12, S52.5--A, S52.5--B, S52.5--C, S52.6--A, S52.6--B, S52.6--C, M80.031A, M80.032A, M80.039A, M84.431A, M84.432A, M84.433A, M84.434A, M84.439A |
| **Co-medications** | **Anatomical Therapeutic Chemical (ATC) codes** |
| Alpha blocker | C02CA |
| Anti-acids | A02B |
| Anti-arrhythmic | C01B |
| Anti-dementia | N06D |
| Anti-depressant (excluding trazodone 'N06AX05') | N06A, N06C |
| Anti-gout | M04 |
| Anti-histamine | R06 |
| Anti-Parkinson | N04 |
| Anti-platelet | B01AC |
| Anti-psychotics (excluding quetiapine 'N05AH04') | N05A |
| Anti-thrombotic | B01AA |
| Benzodiazepines | N05B, N05C |
| Beta blocker | C07 |
| Bronchodilators | R03 |
| Calcitonin | H05BA |
| Calcium channel blockers | C08 |
| Diuretic | C03 |
| Hormone replacement therapy | G03C, G03D, G03F |
| Hypoglycemic agents | A10 |
| Lipid lowering agents | C10 |
| Non-steroidal anti-inflammatory drugs | M01A |
| Propulsive agents | A03FA |
| Quetiapine | N05AH04 |
| Raloxifene | G03XC01 |
| Renin-angiotensin system - acting agents | C09 |
| Oral or intravenous bisphosphonate | M05BA, M05BB |
| Teriparatide | H05AA02 |
| Thiazide | C03A |

© 2024 Huang HK et al. *JAMA Network Open*.

**eTable 2.** Baseline characteristics of the denosumab treatment and comparison groups in the original population without propensity score matching

| | Treatment group (N = 66399) | Comparison group (N = 34897) | Standardized difference |
|---|---|---|---|
| Age, mean years (*SD*) | 76.0 (9.7) | 77.8 (9.9) | -0.18 |
| Sex, n (%) | | | -0.09 |
| Male | 8628 (13.0) | 5605 (16.1) | |
| Female | 57771 (87.0) | 29292 (83.9) | |
| Year of index date, n (%) | | | 0.06 |
| 2012 - 2014 | 8324 (12.5) | 4229 (12.1) | |
| 2015 - 2017 | 21906 (33.0) | 12434 (35.6) | |
| 2018 - 2020 | 36169 (54.5) | 18234 (52.3) | |
| Income (NTD), n (%) | | | 0.12 |
| ≥ 25000 | 20623 (31.1) | 8977 (25.7) | |
| 15000–24999 | 29557 (44.5) | 17457 (50.0) | |
| ≤ 14999 | 16219 (24.4) | 8463 (24.3) | |
| Urban level of residence, n (%) | | | 0.44 |
| 1 (most urban) | 13128 (19.8) | 6032 (17.3) | |
| 2 | 11031 (16.6) | 5594 (16.0) | |
| 3 | 5031 (7.6) | 2615 (7.5) | |
| 4 | 1863 (2.8) | 1195 (3.4) | |
| 5 | 34041 (51.3) | 18450 (52.9) | |
| 6 | 1098 (1.7) | 820 (2.4) | |
| 7 (least urban) | 207 (0.3) | 191 (0.6) | |
| Location of residence in Taiwan, n (%) | | | 0.16 |
| Northern | 25456 (38.3) | 12189 (34.9) | |
| Central | 11270 (17.0) | 6196 (17.8) | |
| Southern | 24046 (36.2) | 13489 (38.7) | |
| Eastern | 5239 (7.9) | 2807 (8.0) | |
| Offshore islands | 388 (0.6) | 216 (0.6) | |
| Healthcare utilization, mean (*SD*) | | | |
| Emergency room visits | 0.79 (1.6) | 1.08 (2.1) | -0.16 |
| Outpatient visits | 43.02 (24.7) | 40.16 (25.1) | 0.11 |
| Hospitalizations | 0.57 (0.9) | 0.8 (1.3) | -0.20 |
| Fracture history, n (%) | | | |
| Hip fracture | 9069 (13.7) | 5198 (14.9) | -0.04 |
| Vertebral fracture | 34462 (51.9) | 17751 (50.9) | 0.02 |
| Wrist or humerus fracture | 2936 (4.4) | 1729 (5.0) | -0.03 |
| Comorbidities, n (%) | | | |
| Asthma | 9182 (13.8) | 4997 (14.3) | -0.01 |
| Cataract | 14515 (21.9) | 6548 (18.8) | 0.08 |
| Congestive heart failure | 4333 (6.5) | 3058 (8.8) | -0.08 |
| COPD | 10836 (16.3) | 6331 (18.1) | -0.05 |
| Dementia | 6404 (9.6) | 4443 (12.7) | -0.10 |
| Depression | 2886 (4.4) | 1615 (4.6) | -0.01 |
| Dyslipidemia | 18100 (27.3) | 8310 (23.8) | 0.08 |
| Gastrointestinal bleeding | 2470 (3.7) | 1625 (4.7) | -0.05 |
| Glaucoma | 4117 (6.2) | 1858 (5.3) | 0.04 |
| Gout | 6705 (10.1) | 4164 (11.9) | -0.06 |

© 2024 Huang HK et al. *JAMA Network Open*.

| | | | |
|---|---|---|---|
| Hemorrhagic stroke | 930 (1.4) | 713 (2.0) | -0.05 |
| Hypertension | 33426 (50.3) | 18770 (53.8) | -0.07 |
| Inflammatory bowel disease | 182 (0.3) | 82 (0.2) | 0.01 |
| Ischemic heart disease | 11219 (16.9) | 6563 (18.8) | -0.05 |
| Ischemic stroke | 3526 (5.3) | 2343 (6.7) | -0.06 |
| Macular degeneration | 3689 (5.6) | 1774 (5.1) | 0.02 |
| Multiple sclerosis | 47 (0.1) | 16 (0.1) | 0.01 |
| Osteoarthritis | 32533 (49.0) | 17349 (49.7) | -0.01 |
| Parkinson disease | 3035 (4.6) | 1854 (5.3) | -0.03 |
| Peptic ulcer | 15444 (23.3) | 8637 (24.8) | -0.03 |
| Pneumonia | 4952 (7.5) | 3644 (10.4) | -0.10 |
| Renal failure | 5976 (9.0) | 3794 (10.9) | -0.06 |
| Rheumatoid arthritis | 3941 (5.9) | 1964 (5.6) | 0.01 |
| Sarcoidosis | 18 (0.0) | 11 (0.0) | -0.00 |
| Schizophrenia | 225 (0.3) | 163 (0.5) | -0.02 |
| Systemic Lupus Erythematosus | 836 (1.3) | 338 (1.0) | 0.03 |
| Previous osteoporosis medication, n (%) | | | |
| Oral or intravenous bisphosphonate | 17045 (25.7) | 8464 (24.3) | 0.03 |
| Teriparatide | 2695 (4.1) | 1595 (4.6) | -0.03 |
| Calcitonin | 1903 (2.9) | 1155 (3.3) | -0.03 |
| Raloxifene | 5777 (8.7) | 2860 (8.2) | 0.02 |
| Baseline medications, n (%) | | | |
| Alpha blocker | 1870 (2.8) | 1113 (3.2) | -0.02 |
| Anti-acids | 36075 (54.3) | 20332 (58.3) | -0.08 |
| Anti-arrhythmic | 5474 (8.2) | 3194 (9.2) | -0.03 |
| Anti-dementia | 5275 (7.9) | 2975 (8.5) | -0.02 |
| Anti-depressant | 12429 (18.7) | 7183 (20.6) | -0.05 |
| Anti-gout | 5222 (7.9) | 3465 (9.9) | -0.07 |
| Anti-histamine | 44149 (66.5) | 23426 (67.1) | -0.01 |
| Anti-Parkinson | 4934 (7.4) | 2958 (8.5) | -0.04 |
| Anti-platelet | 19019 (28.6) | 11246 (32.2) | -0.08 |
| Anti-psychotics | 10185 (15.3) | 6388 (18.3) | -0.08 |
| Anti-thrombotic | 1243 (1.9) | 685 (2.0) | -0.01 |
| Benzodiazepines | 41667 (62.8) | 22976 (65.8) | -0.06 |
| Beta blocker | 20392 (30.7) | 11151 (32.0) | -0.03 |
| Bronchodilators | 27798 (41.9) | 15348 (44.0) | -0.04 |
| Calcium channel blockers | 26829 (40.4) | 15710 (45.0) | -0.09 |
| Diuretic | 14671 (22.1) | 9767 (28.0) | -0.14 |
| Hormone replacement therapy | 1839 (2.8) | 737 (2.1) | 0.04 |
| Lipid lowering agents | 17072 (25.7) | 8009 (23.0) | 0.06 |
| NSAID | 58744 (88.5) | 31512 (90.3) | -0.06 |
| Propulsive agents | 29791 (44.9) | 17040 (48.8) | -0.08 |
| Quetiapine | 4269 (6.4) | 3265 (9.4) | -0.11 |
| RAS-acting agents | 25542 (38.5) | 14454 (41.4) | -0.06 |

COPD: Chronic Obstructive Pulmonary Disease; IQR: interquartile range; NTD: New Taiwan Dollar; NSAID: non-steroidal anti-inflammatory drugs; RAS: renin-angiotensin system; SD: standard deviation.

© 2024 Huang HK et al. *JAMA Network Open*.

**eTable 3.** Detailed results of the analyses stratified by age, sex, and comorbidities

| | Event no. | Person-years | Incidence rate [a] | HR (95% CI) [b] |
|---|---|---|---|---|
| Age < 65 years | | | | |
| Treatment group | 148 | 6125.5 | 24.2 | 1.02 (0.83-1.27) |
| Comparison group | 201 | 8928.3 | 22.5 | 1 (ref.) |
| Age ≥ 65 years | | | | |
| Treatment group | 1868 | 50059.1 | 37.3 | 0.8 (0.75-0.85) |
| Comparison group | 3019 | 64956.7 | 46.5 | 1 (ref.) |
| Males | | | | |
| Treatment group | 317 | 7509.1 | 42.2 | 0.85 (0.73-0.97) |
| Comparison group | 524 | 10456.8 | 50.1 | 1 (ref.) |
| Females | | | | |
| Treatment group | 1699 | 48675.6 | 34.9 | 0.81 (0.76-0.86) |
| Comparison group | 2696 | 63428.2 | 42.5 | 1 (ref.) |
| Dyslipidemia | | | | |
| Treatment group | 531 | 13643.6 | 38.9 | 0.82 (0.73-0.91) |
| Comparison group | 816 | 17450.7 | 46.8 | 1 (ref.) |
| No dyslipidemia | | | | |
| Treatment group | 1485 | 42541.0 | 34.9 | 0.81 (0.76-0.87) |
| Comparison group | 2404 | 56434.3 | 42.6 | 1 (ref.) |
| Hypertension | | | | |
| Treatment group | 1280 | 30260.1 | 42.3 | 0.79 (0.74-0.85) |
| Comparison group | 2103 | 40003.3 | 52.6 | 1 (ref.) |
| No hypertension | | | | |
| Treatment group | 736 | 25924.5 | 28.4 | 0.86 (0.78-0.94) |
| Comparison group | 1117 | 33881.7 | 33.0 | 1 (ref.) |
| Ischemic heart disease | | | | |
| Treatment group | 496 | 10393.0 | 47.7 | 0.82 (0.73-0.92) |
| Comparison group | 753 | 13054.7 | 57.7 | 1 (ref.) |
| No ischemic heart disease | | | | |
| Treatment group | 1520 | 45791.6 | 33.2 | 0.81 (0.76-0.86) |
| Comparison group | 2467 | 60830.2 | 40.6 | 1 (ref.) |
| Renal failure | | | | |
| Treatment group | 368 | 5139.6 | 71.6 | 0.85 (0.74-0.97) |
| Comparison group | 546 | 6695.2 | 81.6 | 1 (ref.) |
| No renal failure | | | | |
| Treatment group | 1648 | 51045.0 | 32.3 | 0.81 (0.76-0.86) |
| Comparison group | 2674 | 67189.7 | 39.8 | 1 (ref.) |

CI: confidence interval; HR: hazard ratio; no.: number; ref.: reference.
[a]Per 1000 person-years.
[b]HR was calculated by Cox proportional hazards model in the propensity score-matched population

© 2024 Huang HK et al. *JAMA Network Open*.

**eTable 4.** Results of the negative control outcome analyses

| | N | Event no. | Person-years | Incidence rate [a] | HR (95% CI) [b] |
|---|---|---|---|---|---|
| Diagnosis of asthma | | | | | |
| Treatment group | 40353 | 1716 | 64461.8 | 26.6 | 0.95 (0.89-1.01) |
| Comparison group | 40353 | 2373 | 87398.7 | 27.2 | 1 (ref.) |
| Diagnosis of lung cancer | | | | | |
| Treatment group | 47035 | 326 | 77275.7 | 4.2 | 1.05 (0.90-1.21) |
| Comparison group | 47035 | 418 | 106038.1 | 3.9 | 1 (ref.) |
| Diagnosis of skin cancer | | | | | |
| Treatment group | 47123 | 145 | 77435.3 | 1.9 | 1.04 (0.84-1.30) |
| Comparison group | 47123 | 187 | 106332.9 | 1.8 | 1 (ref.) |
| Antidepressant use | | | | | |
| Treatment group | 36759 | 3865 | 56393.6 | 68.5 | 0.98 (0.94-1.02) |
| Comparison group | 36759 | 5009 | 74842.4 | 66.9 | 1 (ref.) |
| Thiazide use | | | | | |
| Treatment group | 44767 | 1513 | 71883.4 | 21.0 | 0.97 (0.91-1.04) |
| Comparison group | 44767 | 2043 | 97518.0 | 20.9 | 1 (ref.) |

DM: diabetes mellitus; CI: confidence interval; HR: hazard ratio; no.: number; ref: reference.
[a] Per 1000 person-years.
[b] HR was calculated by the Cox proportional hazards model in the propensity score-matched population

© 2024 Huang HK et al. *JAMA Network Open*.

**eTable 5.** Reasons for end of follow-up or censoring in the treatment and comparison groups in the primary analysis

| | Overall (N = 68,510) | Treatment group (N = 34,255) | Comparison group (N = 34,255) |
|---|---|---|---|
| Reasons, N (%) | | | |
| Development of study outcome | 5236 (7.6) | 2016 (5.9) | 3220 (9.4) |
| Anti-osteoporosis drug initiation | 6624 (9.7) | NA | 6624 (19.3) |
| Denosumab treatment cessation | 17706 (25.8) | 17706 (51.7) | NA |
| Death | 5514 (8.0) | 1680 (4.9) | 3834 (11.2) |
| Data source end date | 33430 (48.8) | 12853 (37.5) | 20577 (60.1) |

NA: not applicable

© 2024 Huang HK et al. *JAMA Network Open*.
