# Supplementary material for: Denosumab and the Risk of Diabetes in Patients Treated for Osteoporosis
Source: JAMA Netw Open. 2024 Feb 9;7(2):e2354734. doi: 10.1001/jamanetworkopen.2023.54734 (PMC10858399; doi:10.1001/jamanetworkopen.2023.54734)
Supplement: Supplement 2. — Data Sharing Statement [file jamanetwopen-e2354734-s002.pdf]

Data Sharing Statement

Huang. Denosumab and the Risk of Diabetes Mellitus in Patients Treated for Osteoporosis. *JAMA Netw Open*. Published February 08, 2024. doi:10.1001/jamanetworkopen.2023.54734

Data
Data available: No

Additional Information
Explanation for why data not available: The datasets generated or analyzed in this study are not publicly accessible due to the data protection policy of the National Health Insurance Research Database (https://nhird.nhri.org.tw/en/Data_Protection.html). Researchers wishing to analyze these datasets must submit a formal application to the Taiwan Ministry of Health and Welfare for access. For more information, please visit their website at https://dep.mohw.gov.tw/DOS/cp-5119-59201-113.html.
